# Clinical features of early-onset type 2 diabetes and its association with triglyceride glucose-body mass index: a cross-sectional study

**DOI:** 10.3389/fendo.2024.1356942

**Published:** 2024-03-11

**Authors:** Yanjuan Jiang, Xiaoyang Lai

**Affiliations:** The Second Affiliated Hospital, Jiangxi Medical College, Nanchang University, Nanchang, China

**Keywords:** early-onset type 2 diabetes, insulin resistance, overweight, obesity, triglyceride glucose-body mass index

## Abstract

**Objective:**

The incidence of early-onset type 2 diabetes (T2D) has increased significantly, with insulin resistance (IR) and obesity being the main drivers of its onset. This study aims to investigate the clinical characteristics of early-onset T2D and its association with triglyceride glucose body mass index (TyG-BMI), an emerging surrogate of IR.

**Methods:**

A total of 1000 adults newly diagnosed with T2D were enrolled and divided into early-onset T2D (18~40 years, N=500) and late-onset T2D groups (≥40 years, N=500). Independent *t* and chi-squared tests were used to compare the characteristics of the two groups, and logistic regression analysis, trend tests, restricted cubic spline curves (RCSs), and receiver operating characteristic (ROC) curves were used to identify the relationship between TyG-BMI and early-onset T2D.

**Results:**

Patients with early-onset T2D were more likely to have a higher body mass index (BMI), hemoglobin A1C (HbA_1c_), fasting plasma glucose (FPG), total cholesterol (TC), triglycerides (TG), low-density lipoprotein cholesterol (LDL-C), serum uric acid (SUA), triglyceride glucose index (TyG), and TyG-BMI (*P* < 0.05). A higher TyG-BMI was associated with an increased risk of early-onset T2D (*P* < 0.001). The RCSs showed a nonlinear relationship between TyG-BMI and early-onset T2D, and the slope of the curve increased with an increase in TyG-BMI (*P* for nonlinearity < 0.001). In the subgroup analysis, additive interactions between TyG-BMI and the risk of early-onset T2D were observed for sex, family history of diabetes, BMI, fatty liver, and hypertension (*P* < 0.001). ROC curve showed that the area under the curve of TyG-BMI was 0.6781, which was larger than its main components (TyG, BMI, FPG, TG). The best cutoff value was 254.865, the sensitivity was 74.6%, and the specificity was 53.6%.

**Conclusion:**

Patients with early-onset T2D are characterized by severe IR, metabolic disorders, and being overweight/obese and an increase in TyG-BMI is independently associated with an increased risk of early-onset T2D.

## 1 Introduction

The global prevalence of diabetes and obesity continues to rise, as does the incidence of early-onset type 2 diabetes (T2D), which has dramatically accelerated in the wake of the COVID-19 pandemic (1, 2). A systematic review reported that during 1990 to 2019, the early-onset of T2D in the world (here defined as 15 to 39 years) age-standardized prevalence rates increased from of 117.22/100,000 to 183.36/100,000, disability-adjusted life-years rate increased from 106.34/100,000 to 149.61/100,000, and the age-standardized mortality rate in 2019 was 0.77/100, 000(0.76-0.78) (3). Early-onset T2D is more aggressive, with a more rapid course of disease deterioration and beta-cell failure compared with late-onset T2D or type 1 diabetes (T1D) (4). Due to the longer duration of exposure to hyperglycemia, patients with early-onset T2D are at a significantly increased risk of microvascular and macrovascular complications, dementia, and tumors, and a decreased quality of life and life expectancy, which seriously increases the burden on patients, families, and society (5–13).

Current evidence shows that the pathogenesis of early-onset T2D is similar to that of late-onset T2D, mainly including β-cell dysfunction, insulin resistance (IR) and obesity (14–16). Several studies suggest that early-onset visceral obesity and associated insulin resistance may be major risk factors for the early progression of T2D (10, 15, 17). A Danish study found that obesity contributes to risk for T2D significantly more than genetic factors or poor lifestyle (18). The triglyceride-glucose index (TyG), based on triglycerides (TG) and fasting plasma glucose (FPG), has been widely established as an excellent index for evaluating IR (19, 20). Recently, a study focused on the triglyceride glucose-body mass index (TyG-BMI), a derivative of body mass index (BMI) and TyG, which can simultaneously capture BMI, blood glucose and lipid profiles, and more closely reflect IR than TyG (21). Additionally, TyG-BMI has good predictive performance in hypertension, metabolic syndrome, nonalcoholic fatty liver disease, prediabetes, and diabetes (22–28). Since early-onset T2D is closely associated with IR and obesity, we hypothesized that TyG-BMI may be a useful predictor of early-onset T2D.

## 2 Methods

### 2.1 Study participants

In this cross-sectional study, we enrolled 1000 patients with newly diagnosed T2D who were admitted to the Department of Endocrinology of the Second Affiliated Hospital of Nanchang University between March 2022 and September 2023. Patients were included in the study cohort if they: (1) met the diagnostic criteria of World Health Organization Diabetes Experts Committee in 1999 for diabetes and HbA_1c_ ≥ 6.5%; (2) age of onset ≥ 18 years old; (3) none of the patients received hypoglycemic drugs within 3 months before admission. The exclusion criteria were as follows: (1) T1D, special types of diabetes, gestational diabetes, and uncertain types of diabetes; (2) diabetes during pregnancy or lactation; and (3) severe organ dysfunction, tumor, or severe infection. According to previous studies, all participants were divided into an early-onset T2D group (< 40 years old) and a late-onset T2D group (≥ 40 years old). This study was approved by the Ethics Committee of the Second Affiliated Hospital of Nanchang University (No. IIT-O-2023-122) and performed in accordance with the principles of the Declaration of Helsinki, revised in 2008. Informed consent was obtained from all participants.

### 2.2 Data collection and measurement

All participants completed a standardized admission questionnaire containing demographic characteristics, medical history, and lifestyle information under the guidance of a medical worker. Height, weight, and blood pressure were measured and recorded by nurses. Blood pressure was measured using a standard mercury sphygmograph, and all participants were asked to rest for at least 5 minutes in a quiet environment before blood pressure was measured. All participants were required to fast for at least 10 h. Venous blood was drawn from the elbow in the morning and 2 h after breakfast, and midstream urine samples were collected in the morning. Hemoglobin A1c (HbA_1c_), serum glucose, serum C-peptide, total cholesterol (TC), TG, low-density lipoprotein cholesterol (LDL-C), high-density lipoprotein cholesterol (HDL-C), serum uric acid (SUA), serum creatinine (Scr), and urinary albumin-to-creatinine ratio (UACR) were measured using an automatic biochemical and immunological analyzer (cobas 8000, Roche, Germany). The estimated glomerular filtration rate (eGFR) was calculated using the CRIC equation. All the samples were tested in the same laboratory. Comorbidities such as hypertension, fatty liver disease, coronary heart disease, and stroke were all diagnosed by medical institutions or had direct laboratory test evidence.

### 2.3 Definition and *calculation*

The diagnosis of early-onset T2D in adults was excluded; that is, patients aged 18-40 years could be diagnosed with early-onset T2D in adults after excluding those with T1D, monogenic diabetes, secondary diabetes, gestational diabetes, and no characteristics of undetermined diabetes. Diagnostic age was defined according to the American Diabetes Association (ADA) and World Health Organization (WHO) criteria (9).

The formulae for calculating BMI, TyG, and TyG-BMI are as follows: BMI = body weight (kg)/height (m^2^), TyG = ln (fasting triglycerides [mg/dL] × FPG [mg/dL]/2), and TyG-BMI = TyG × BMI (19, 29).

### 2.4 Statistical analysis

All data analyses and graphics were performed using SPSS 26.0 and R language 4.3.1. Participants’ measurement data were tested for normality. The normal distribution was expressed as the mean ± standard deviation, the non-normal distribution was expressed as the median and interquartile range (M[QL, QU]), and the count data was expressed as the number of cases and percentage. The independent sample t-test and Mann-Whitney U test were used to compare the characteristics of continuous variables between the two groups, and one-way analysis of variance and the Kruskal-Wallis rank sum test were used to compare the differences among multiple groups. Differences in characteristics between groups for categorical variables were analyzed using the chi-square and fisher’s exact tests.

BMI, TyG, and TyG-BMI were grouped into quartiles, and logistic regression analysis was used to evaluate the relationship between the risk of early-onset T2D and the three indicators. We reported the odds ratio (OR) and 95% confidence interval (CI) and tested the trend using the first quartile as a reference. We used restricted cubic spline curves (RCSs) to identify the nonlinear associations of BMI, TyG, and TyG-BMI with the risk of early-onset T2D and to explore the nonlinear associations of TyG-BMI with the risk of early-onset T2D from the perspective of sex. The receiver operating characteristic (ROC) curve was used to describe the diagnostic effect of FPG, TG, BMI, TyG, and TyG-BMI on early-onset T2D, and the area under the ROC curve (AUC) was used to evaluate the diagnostic value of each. Subgroup analyses were conducted according to sex, family history of diabetes, body mass index (BMI), smoking, alcohol consumption, fatty liver, and hypertension. BMI was stratified according to the standard recommended by the Working Group on Obesity in China, with 24kg/m^2^ as the cut-off point for overweight (30). *P* < 0.05 was considered statistically significant.

## 3 Results

### 3.1 Study population

**Figure 1:**
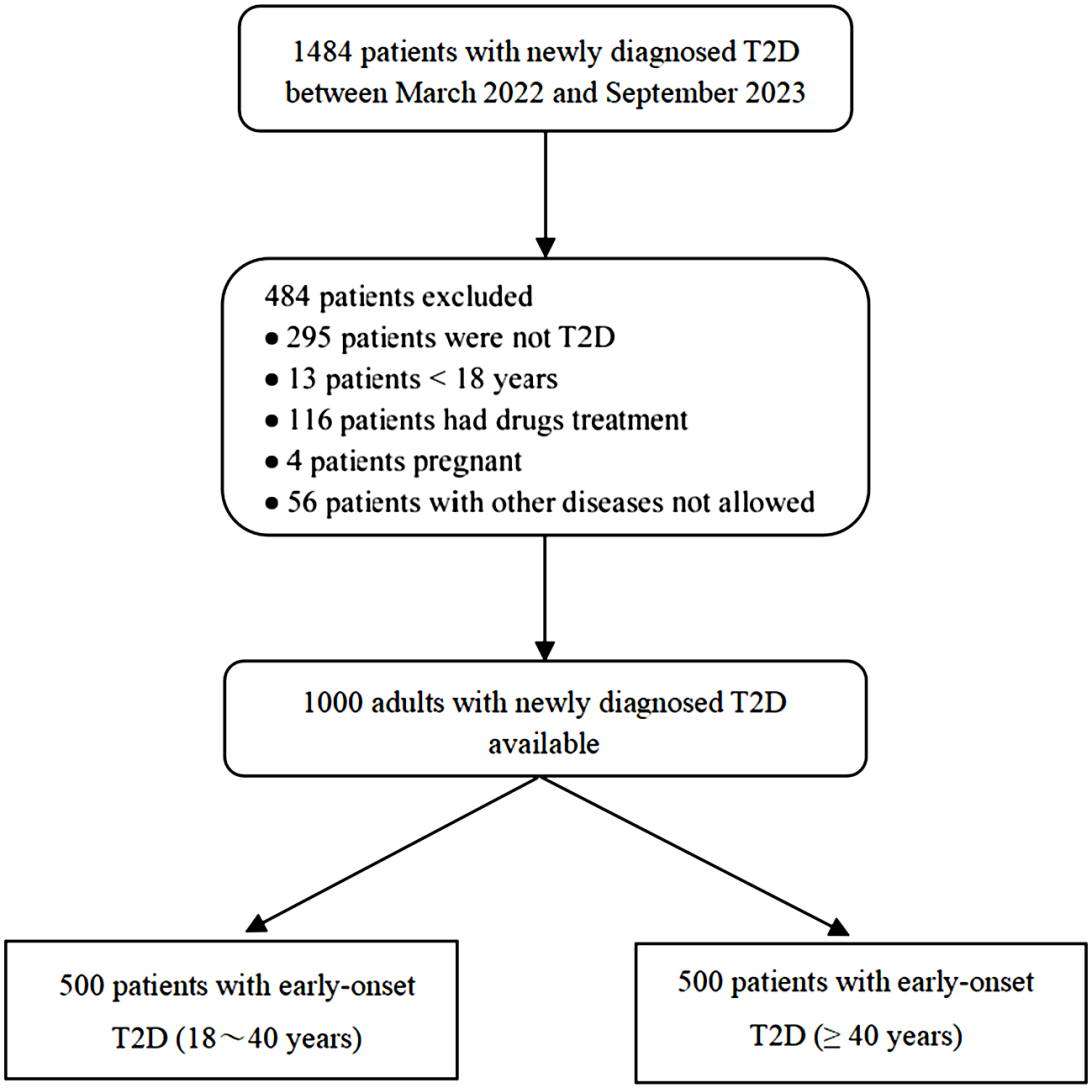
The flow chart of participants selection.

We consecutively recruited 1484 patients from the Department of Endocrinology of the Second Affiliated Hospital of Nanchang University between March 2022 and September 2023. Based on the inclusion criteria, 484 patients were excluded. Details of participant screening are shown in **Figure 1**.

### 3.2 Clinical characteristics of patients with early-onset T2D

Compared with the late-onset T2D group, the proportion of male and family history of diabetes was higher in the early-onset T2D group (*P* < 0.05). In the early stage of the disease course, patients with early-onset T2D had a larger average body weight and higher BMI and tended to have lower systolic blood pressure and higher diastolic blood pressure (*P* < 0.05). The levels of HbA1c, FPG, 2-hour postprandial plasma glucose (2hPG), 2hC-P, TC, TG, LDL-C, SUA, eGFR, TyG, and TyG-BMI were higher and the levels of HDL-C and Scr were lower in the early-onset T2D group (*P* < 0.05). In addition, the prevalence of fatty liver in the early-onset T2D group was higher than that in the late-onset T2D group (57.6% vs. 46.0%), but the prevalence of hypertension (12.4% vs. 55.4%), coronary heart disease (1.8% vs. 20.6%), and stroke (0.4%vs5.0%) was significantly lower than that in the late-onset T2D group (*P* < 0.05) (**Table 1**).

**Table 1:** The baseline characteristics of participants.

Characteristics	Total (n=1000)	Early-onset T2D (n = 500)	Late-onset T2D (n = 500)	*P*, Value
Age, years	40 (33, 56)	33 (28, 37)	56 (49, 64)	0.000^*^
Gender, n(%)				0.000^*^
Male	697 (69.7%)	385 (77.0%)	312 (62.4%)	
Female	303 (30.3%)	115 (23.0%)	188 (37.6%)	
Family history of diabetes, n(%)				0.000^*^
Yes	176 (17.6%)	112 (22.4%)	64 (12.8%)	
No	824 (82.4%)	388 (77.6%)	436 (87.2%)	
Smoking, n(%)				0.279
Yes	175 (17.5%)	81 (16.2%)	94 (18.8%)	
No	825 (82.5%)	419 (83.8%)	406 (81.2%)	
Yes	144 (14.4%)	69 (13.8%)	75 (15.0%)	
No	856 (85.6%)	431 (86.2%)	425 (85.0%)	
Height, m	1.66 ± 0.08	1.69 ± 0.80	1.64 ± 0.82	0.000^*^
Weight, kg	69.48 ± 14.50	74.29 ± 15.69	64.68 ± 11.35	0.000^*^
BMI, kg/m^2^	26.03 ± 4.46	26.03 ± 4.46	24.00 ± 3.45	0.000^*^
SBP, mmHg	126.88 ± 26.65	124.13 ± 16.14	129.64 ± 33.86	0.001^*^
DBP, mmHg	83.96 ± 11.60	83.96 ± 11.60	81.82 ± 11.75	0.004^*^
HbA_1c_, %	10.19 ± 2.72	10.80 ± 2.55	9.59 ± 2.75	0.000^*^
FPG, mmol/L	11.48 (7.59, 15.15)	12.67 (8.46, 15.89)	9.53 (6.73, 14.34)	0.000^*^
2hPG, mmol/L	16.60 (12.10, 21.95)	18.10 (13.43, 22.41)	15.04 (11.20, 21.36)	0.000^*^
FC-P, ng/mL	2.02 (1.13, 2.81)	2.06 (1.20, 2.84)	1.93 (1.08, 2.79)	0.532
2hC-P, ng/mL	3.85 (2.04, 5.44)	3.76 (1.93, 4.95)	3.92 (2.18, 6.30)	0.004^*^
TC, mmol/L	5.29 ± 1.64	5.52 ± 1.77	5.05 ± 1.45	0.000^*^
TG, mmol/L	1.88 (1.16, 3.26)	2.24 (1.38, 3.99)	1.54 (1.03, 2.51)	0.000^*^
LDL-C, mmol/L	3.18 ± 1.13	3.30 ± 1.12	3.06 ± 1.13	0.001^*^
HDL-C, mmol/L	1.07 ± 0.33	0.99 ± 0.30	1.14 ± 0.33	0.000^*^
SUA, μmol/L	357.93 ± 117.51	380.01 ± 121.92	335.85 ± 108.64	0.000^*^
Scr, μmol/L	64.22 (54.17, 75.37)	62.70 (53.72, 72.17)	66.50 (54.97, 80.22)	0.000^*^
UACR, mg/g	11.35 (4.92, 31.87)	83.44 ± 402.86	63.65± 185.00	0.168
eGFR, ml/mim/(1.73m^2^)	116.99 ± 31.70	129.93 ± 22.41	104.05 ± 34.26	0.000^*^
TyG	9.77 ± 1.05	10.04 ± 1.08	9.50 ± 0.95	0.000^*^
TyG-BMI	245.48 ± 53.40	262.37 ± 57.60	228.60 ± 42.66	0.000^*^
Fatty liver, n(%)				0.000^*^
Yes	518 (51.8%)	288 (57.6%)	230 (46.0%)	
No	482 (48.2%)	212 (42.4%)	270 (54.0%)	
Hypertension, n(%)				0.000^*^
Yes	339 (33.9%)	62 (12.4%)	277 (55.4%)	
No	661 (66.1%)	438 (87.6%)	223 (44.6%)	
CHD, n(%)				0.000^*^
Yes	112 (11.2%)	9 (1.8%)	103 (20.6%)	
No	888 (88.8%)	491 (98.2%)	397 (79.4%)	
Stroke, n(%)				0.000^*^
Yes	27 (2.7%)	2 (0.4%)	25 (5.0%)	
No	973 (97.3%)	498 (99.6%)	475 (95.0%)	

Data are presented as mean ± SD, interquartile range, percentage; comparisons between the two groups are performed using t-test or χ^2^ test, ^*^ *P*<0.05.

T2D, type 2 diabetes; BMI, Body mass index; SBP, Systolic blood pressure; DBP, Diastolic blood pressure; HbA_1c_, Hemoglobin A1C; FPG, Fasting plasma glucose; 2hPG, 2-hours postprandial blood glucose; FC-P, Fasting C-peptide; 2hC-P 2 hours postprandial C-peptide; TC, Total cholesterol; TG, Triglycerides; LDL-C, Low-density lipoprotein cholesterol; HDL-C, High-density lipoprotein cholesterol; SUA, Serum uric acid, Scr, serum creatinine; UACR, urinary albumin to creatinine ratio; eGFR, estimated glomerular filtration rate; TyG, triglyceride glucose index; TyG-BMI, triglyceride glucose-body mass index; CHD, coronary heart disease.

### 3.3 The multivariate logistic regression for early-onset T2D

**Figure 2:**
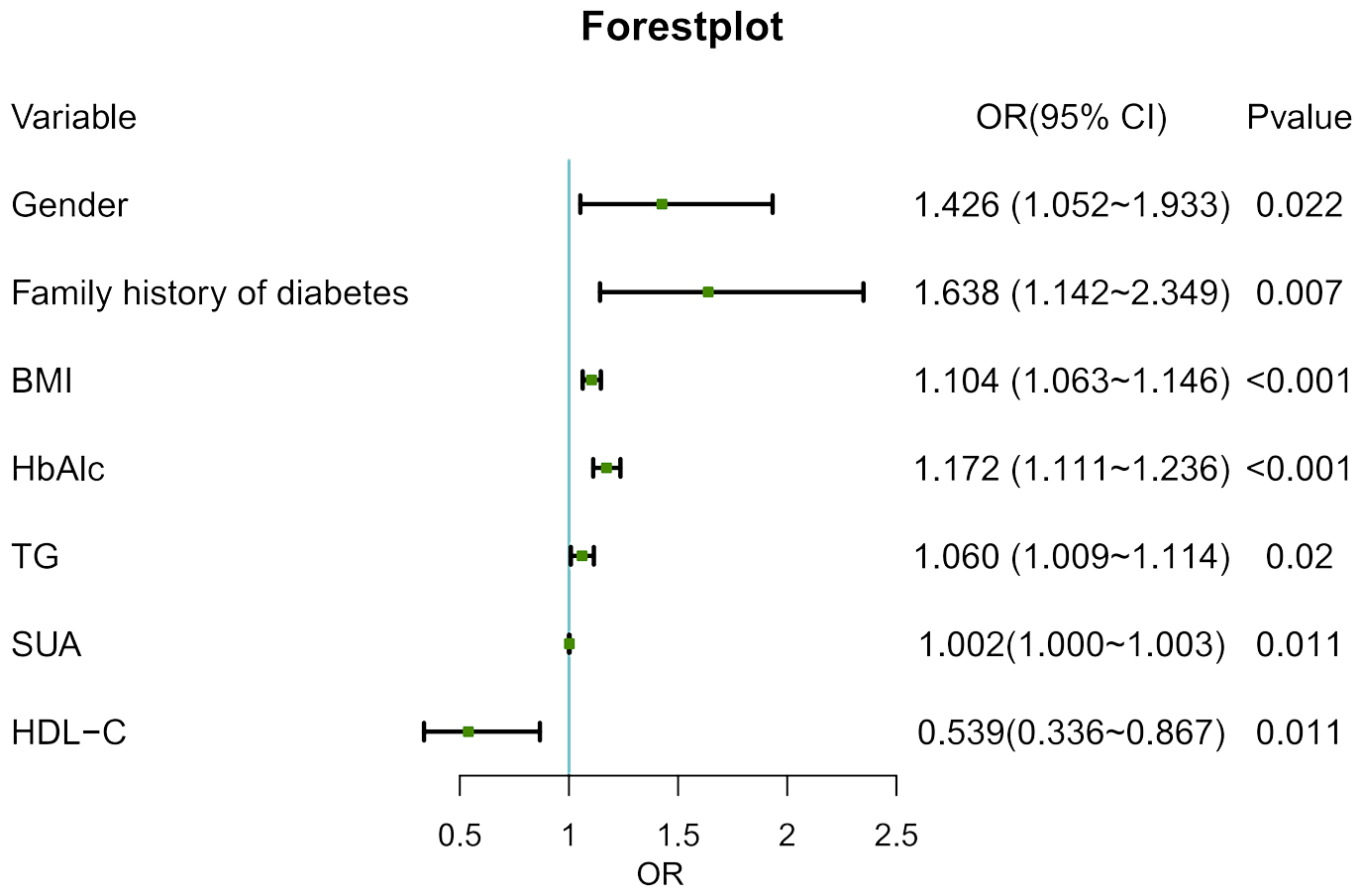
Forestplot of risk factors for early-onset T2D.

With age at onset as the dependent variable and excluding confounding factors, the statistically significant indicators in **Table 1** were included in the multivariate logistic regression model as independent variables. The results showed that male, family history of diabetes, BMI, HbA_1c_, TG and SUA may have positive effects on the occurrence of early-onset of T2D, among which family history of diabetes had the greatest influence (*P* < 0.05) (**Figure 2**).

### 3.4 Basic characteristics of the early-onset T2D according to TyG index quartiles

When the enrolled patients with early-onset T2D were grouped according to the TyG-BMI quartile, the results showed that the higher quartile of TyG-BMI was more likely to include younger patients, a higher proportion of men, and had higher body weight, BMI, SBP, and DBP. In terms of metabolic indicators, the higher quartile group of TyG-BMI had higher levels of FPG, 2hPG, TC, TG, LDL-C, and SUA; lower levels of HDL-C; and higher proportions of fatty liver and hypertension (*P* < 0.05) (**Table 2**).

**Table 2:** Basic characteristics of the early-onset T2D according to TyG-BMI quartiles.

Characteristics	TyG-BMI quartile				*P*, Value
	Q1 (115.07-222.22)	Q2 (222.22-257.58)	Q3 (257.58-294.65)	Q4 (294.65-502.83)	
No. of subjects	125	125	125	125	
Age, years	34 (31, 37)	34 (30, 37)	32 (28, 37)	30 (27, 35)	0.000^*^
Gender, n(%)					0.001^*^
Male	83 (66.4%)	91 (72.8%)	107 (85.6%)	104 (83.2%)	
Female	42 (33.6%)	34 (27.2%)	18 (14.4%)	21 (16.8%)	
Family history of diabetes, n(%)					0.217
Yes	20 (16.0%)	29 (23.2%)	33 (26.4%)	30 (24.0%)	
No	105 (84.0%)	96 (76.8%)	92 (73.6%)	95 (76.0%)	
Smoking, n(%)					0.128
Yes	15 (12.0%)	16 (12.8%)	27 (21.6%)	23 (18.4%)	
No	110 (88.0%)	109 (87.2%)	98 (78.4%)	102 (81.6%)	
Alcohol drinking, n(%)					0.273
Yes	13 (10.4%)	14 (11.2%)	22 (17.6%)	20 (16.0%)	
No	112 (89.6%)	111 (88.8%)	103 (82.4%)	105 (84.0%)	
Height, m	1.66 ± 0.08	1.68 ± 0.08	1.70 ± 0.08	1.70 ± 0.07	0.000^*^
Weight, kg	58.93 ± 7.86	69.21 ± 9.33	78.31 ± 9.94	90.69 ± 13.84	0.000^*^
BMI, kg/m^2^	21.42 ± 2.03	24.50 ± 2.18	26.98 ± 2.22	31.21 ± 3.82	0.000^*^
SBP, mmHg	118.83 ± 16.64	122.60 ± 16.20	126.83 ± 15.45	128.24 ± 14.65	0.000^*^
DBP, mmHg	79.58 ± 9.83	83.20 ± 12.42	85.49 ± 11.61	87.58 ± 10.98	0.000^*^
HbA_1c_, %	10.44 ± 2.84	10.92 ± 2.51	10.65 ± 2.51	11.18 ± 2.28	0.347
FPG, mmol/L	9.69 (6.99, 13.91)	12.76 (8.36, 16.29)	12.73 (8.83, 15.86)	14.21 (11.19, 17.54)	0.000^*^
2hPG, mmol/L	16.76 ± 7.13	18.56 ± 7.06	19.04 ± 6.93	19.77 ± 6.37	0.002^*^
FC-P, ng/mL	1.37 (0.80, 2.06)	1.90 (1.06, 2.66)	2.06 (1.52, 2.99)	2.23 (1.66, 3.18)	0.000^*^
2hC-P, ng/mL	2.73 (1.20, 4.28)	3.73 (1.88, 4.68)	3.70 (2.22, 4.96)	4.28 (2.79, 5.42)	0.001^*^
TC, mmol/L	4.77 (4.09, 5.57)	5.03 (4.40, 6.15)	5.42 (4.56, 6.36)	5.88 (5.23, 7.20)	0.000^*^
TG, mmol/L	1.14 (0.82, 1.83)	2.12 (1.37, 3.03)	2.78 (1.78, 5.01)	4.55 (2.82, 8.56)	0.000^*^
LDL-C, mmol/L	2.97 ± 0.96	3.25 ± 1.09	3.41 ± 1.07	3.55 ± 1.29	0.000^*^
HDL-C, mmol/L	1.17 ± 0.35	1.02 ± 0.32	0.93 ± 0.23	0.84 ± 0.20	0.000^*^
SUA, μmol/L	293.79 (252.93, 351.53)	334.52 (279.70, 426.32)	390.89 (321.84, 477.28)	419.31 (358.78, 498.00)	0.000^*^
Scr, μmol/L	59.79 (49.67, 68.63)	62.98 (52.46, 69.95)	63.82 (55.90, 74.93)	63.82 (55.96, 74.43)	0.512
UACR, mg/g	7.48 (3.63, 25.81)	9.75 (5.06, 21.64)	11.38 (4.89, 25.67)	12.66 (6.08, 41.31)	0.904
eGFR, ml/mim/(1.73m^2^)	124.75 (122.29, 137.00)	124.75 (122.27, 136.60)	124.40 (120.70, 136.32)	126.32 (122.25, 137.00)	0.754
TyG	9.13 ± 0.80	9.90 ± 0.88	10.25 ± 0.87	10.90 ± 0.95	0.000^*^
Fatty liver, n(%)					0.000^*^
Yes	30 (24.0%)	74 (59.2%)	89 (71.2%)	95 (76.0%)	
No	95 (76.0%)	51 (40.8%)	36 (28.8%)	30 (24.0%)	
Hypertension, n(%)					0.036^*^
Yes	8 (6.4%)	14 (11.2%)	17 (13.6%)	23 (18.4%)	
No	117 (93.6%)	111 (88.8%)	108 (86.4%)	102 (81.6%)	
CHD, n(%)					0.150
Yes	2 (1.6%)	0 (0.0%)	2 (1.6%)	5 (4.0%)	
No	123 (98.4%)	125 (100%)	123 (98.4%)	120 96.0%)	
Stroke, n(%)					1.000
Yes	0 (0.0%)	1 (0.8%)	1 (0.8%)	0 (0.0%)	
No	125 (100%)	124 (99.2%)	124 (99.2%)	125 (100%)	

Data are presented as mean ± SD, interquartile range, percentage; comparisons between the four groups are performed using one-way analysis of variance and Kruskal-Wallis rank sum test, ^*^ *P*<0.05.

T2D, type 2 diabetes; BMI, Body mass index; SBP, Systolic blood pressure; DBP, Diastolic blood pressure; HbA1c, Hemoglobin A1C; FPG, Fasting plasma glucose; 2hPG, 2-hours postprandial blood glucose; FC-P, Fasting C-peptide; 2hC-P 2 hours postprandial C-peptide; TC, Total cholesterol; TG, Triglycerides; LDL-C, Low-density lipoprotein cholesterol; HDL-C, High-density lipoprotein cholesterol; SUA, Serum uric acid, Scr, serum creatinine; UACR, urinary albumin to creatinine ratio; eGFR, estimated glomerular filtration rate; TyG, triglyceride glucose index; TyG-BMI, triglyceride glucose-body mass index; CHD, coronary heart disease.

### 3.5 Logistic regression analysis of the relationship between TyG-BMI and early-onset T2D

**Table 3** shows the association of BMI, TyG, and TyG-BMI with early-onset T2D using multivariate logistic regression models. In addition to the crude model, two models were constructed and adjusted according to the results of the univariate analysis and potential confounders selected from literature reports. Model 1 was adjusted for sex and family history of diabetes, and Model 2 was adjusted for SBP, DBP, HbA_1c_, TC, LDL-C, HDL-C, and SUA levels based on Model 1. The results showed that in Model 3, when TyG-BMI was considered as a continuous variable, the risk of early-onset T2D increased by 9% for every 1-unit increase in TyG-BMI (OR = 1.009, 95%CI 1.006-1.013). When TyG-BMI was evaluated by quartile, using quartile 1 as a reference, a higher TyG-BMI was associated with an increased risk of early-onset T2D (*P* < 0.001). The risk in quartile 4 was significantly higher than that in quartiles 2 and 3, and the OR values in the three models were 5.209(3.553-7.637), 4.781(3.242-7.048), 2.771(1.720-4.467), respectively. In multivariate models 1 and 2, the association between TyG-BMI and early-onset T2D was slightly attenuated, but a positive dose-response relationship was maintained. Therefore, TyG-BMI is an independent risk factor of early-onset T2D.

**Table 3:** Logistic regression analysis of the relationship between TyG-BMI and early-onset T2D.

Parameters	Crude Model OR(95% CI), *p* value	Multivariate Model 1 OR(95% CI), *p* value	Multivariate Model 2 OR(95% CI), *p* value
TyG-BMI	1.014(1.011~1.017), 0.000^*^	1.013(1.010~1.016), 0.000^*^	1.009(1.006~1.013), 0.000^*^
TyG-BMI quartile			
Q1	reference	reference	reference
Q2	1.314(0.914~1.889), 0.140	1.351(0.935~1.953), 0.109	1.154(0.772~1.725), 0.486
Q3	2.033(1.418~2.914), 0.000^*^	1.822(1.262~2.630), 0.001^*^	1.268(0.832~1.933), 0.269
Q4	5.209(3.553~7.637), 0.000^*^	4.781(3.242~7.048), 0.000^*^	2.771(1.720~4.467), 0.000^*^
*P* for trend	0.000^*^	0.000^*^	0.000^*^
TyG	1.690(1.483~1.926), 0.000^*^	1.611(1.411~1.840), 0.000^*^	1.096(0.906~1.325), 0.345
TyG quartile			
Q1	reference	reference	reference
Q2	1.506(1.047~2.165), 0.027^*^	1.466(1.013~2.122), 0.042^*^	0.958(0.635~1.444), 0.837
Q3	2.561(1.782~3.681), 0.000^*^	2.561(1.773~3.699), 0.000^*^	1.409(0.916~2.167), 0.119
Q4	4.276(2.941~6.215), 0.000^*^	3.696(2.525~5.409), 0.000^*^	1.371(1.003~1.874), 0.237
*P* for trend	0.000^*^	0.000^*^	0.083
BMI	1.141(1.102~1.181), 0.000^*^	1.133(1.095~1.173), 0.000^*^	1.117(1.073~1.162), 0.000^*^
BMI quartile			
Q1	reference	reference	reference
Q2	1.327(0.930~1.896), 0.120	1.317(0.916~1.894), 0.137	1.241(0.837~1.840), 0.282
Q3	1.391(0.974~1.988), 0.070	1.329(0.924~1.911), 0.125	1.147(0.763~1.724), 0.510
Q4	3.877(2.671~5.629), 0.000^*^	3.567(2.441~5.211), 0.000^*^	2.853(1.855~4.388), 0.000^*^
*P* for trend	0.000^*^	0.000^*^	0.000^*^

Data are presented as odds ratios (ORs), 95% confidence intervals, (CIs) or P value. Crude Model: Unadjusted; Multivariate Model 1: Adjusted for gender, family history of diabetes; Multivariate Model 2: Adjusted for gender, family history of diabetes, SBP, DBP, HbA1c, TC, LDL-C, HDL-C, SUA.^*^ *P*<0.05.

T2D, type 2 diabetes; BMI, Body mass index; TyG, triglyceride glucose index; TyG-BMI, triglyceride glucose-body mass index.

### 3.6 Nonlinear *relationship* between TyG-BMI and *early*-onset T2D

**Figure 3:**
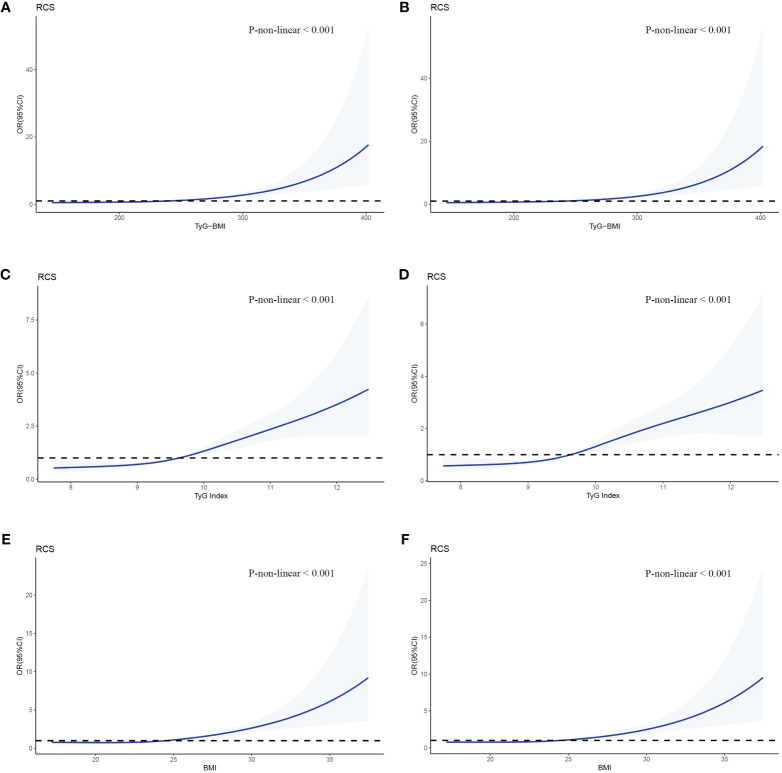
RCSs of the association between TyG-BMI and early-onset T2D. The odd ratios for early-onset T2D (solid line) and 95% confidence intervals (shaded portion) are presented. **(A, B)** Correlation between TyG-BMI and early-onset T2D in the crude model and multivariate model(adjusts for gender and family history of diabetes). **(C, D)** Correlation between TyG and early-onset T2D in the crude model and multivariate model. **(E, F)** Correlation between BMI and early-onset T2D in the crude model and multivariate model.

**Figure 4:**
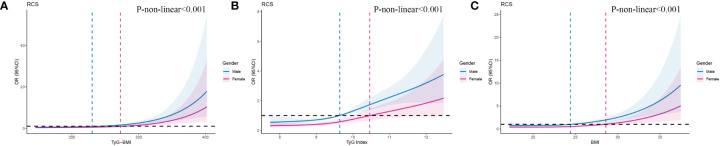
RCSs of the association between TyG-BMl and early-onset T2D according to gender. As shown on the labels, blue line and area indicates male, pink line and area indicates female.

We used RCSs to fit the curve of the relationship between longitudinal changes in TyG-BMI and early-onset T2D. The results showed a nonlinear dose-response relationship between TyG-BMI and the risk of early-onset T2D. The slope of the curve increased with increasing TyG-BMI (*P* for nonlinearity < 0.001) and the inflpoint was 240.82 (**Figure 3A**). This nonlinear dose-response relationship remained stable (*P* for nonlinearity < 0.001) when re-fitting the RCSs after adjusting for covariates (sex and family history of diabetes) (**Figure 3B**). The relationships between TyG and BMI and early-onset T2D were similar to that of TyG-BMI (**Figures 3C–F**). We fitted the RCSs of TyG-BMI and early-onset T2D separately according to sex. The results showed that the relationship was more significant in males, with the inflection points of RCSs of 230.3 and 272.6 for males and females, respectively. The risk of early-onset T2D increased with an increase in TyG-BMI when TyG-BMI exceeded the inflection point value (*P* for nonlinearity < 0.001). BMI and TyG index also showed a similar curve correlation (*P* for nonlinearity < 0.001). The inflection points of RCSs for BMI in men and women were 24.41 and 28.60, respectively, and those for TyG in men and women were 9.64 and 10.46, respectively (**Figure 4**).

### 3.7 Subgroup *analysis*

To further explore the influence of other risk factors on the correlation between TyG-BMI and the risk of early-onset T2D, subgroup analyses were performed according to the following stratification variables: sex, family history of diabetes, BMI, smoking, alcohol consumption, fatty liver, and hypertension. The results of the subgroup analyses and interactions are summarized in **Table 4**. Additive interactions between TyG-BMI and the risk of early-onset T2D were observed for sex, family history of diabetes, BMI, fatty liver disease, and hypertension (*P* < 0.001). Stronger associations were found in participants who were male, had a family history of diabetes, were overweight (24 kg/m^2^ ≤ BMI < 28 kg/m^2^), had fatty liver, and had hypertension. However, no significant interaction was observed between smoking and alcohol consumption.

**Table 4:** Subgroup analysis of the associations between TyG-BMI and the risk of early-onset T2D.

Subgroup	No. of subjects	OR (95%CI)	*P* value	*P* for Interaction
Gender				0.000^*^
Male	697	1.015 (1.011~1.018)	0.000^*^	
Female	303	1.010 (1.005~1.015)	0.000^*^	
Family history of diabetes				0.000^*^
Yes	176	1.014 (1.007~1.022)	0.000^*^	
No	824	1.013 (1.010~1.016)	0.000^*^	
Smoking				0.953
Yes	175	1.017 (1.010~1.025)	0.000^*^	
No	825	1.013 (1.010~1.016)	0.000^*^	
Alcohol drinking				0.640
Yes	144	1.017 (1.009~1.025)	0.000^*^	
No	856	1.013 (1.010~1.017)	0.000^*^	
Fatty liver				0.000^*^
Yes	518	1.019 (1.014~1.024)	0.000^*^	
No	482	1.010 (1.005~1.014)	0.000^*^	
Hypertension				0.000^*^
Yes	339	1.019 (1.013~1.025)	0.000^*^	
No	661	1.014 (1.010~1.018)	0.000^*^	
BMI, kg/m^2^				0.000^*^
<24	435	1.008 (1.002~1.015)	0.015^*^	
≥24, <28	362	1.022 (1.013~1.031)	0.000^*^	
≥28	198	1.017 (1.008~1.026)	0.000^*^	

^*^P<0.05. TyG-BMI, triglyceride glucose-body mass index; T2D, type 2 diabetes; BMI, Body mass index.

### 3.8 Diagnostic *value* of TyG-BMI for *early*-onset T2D

**Figure 5:**
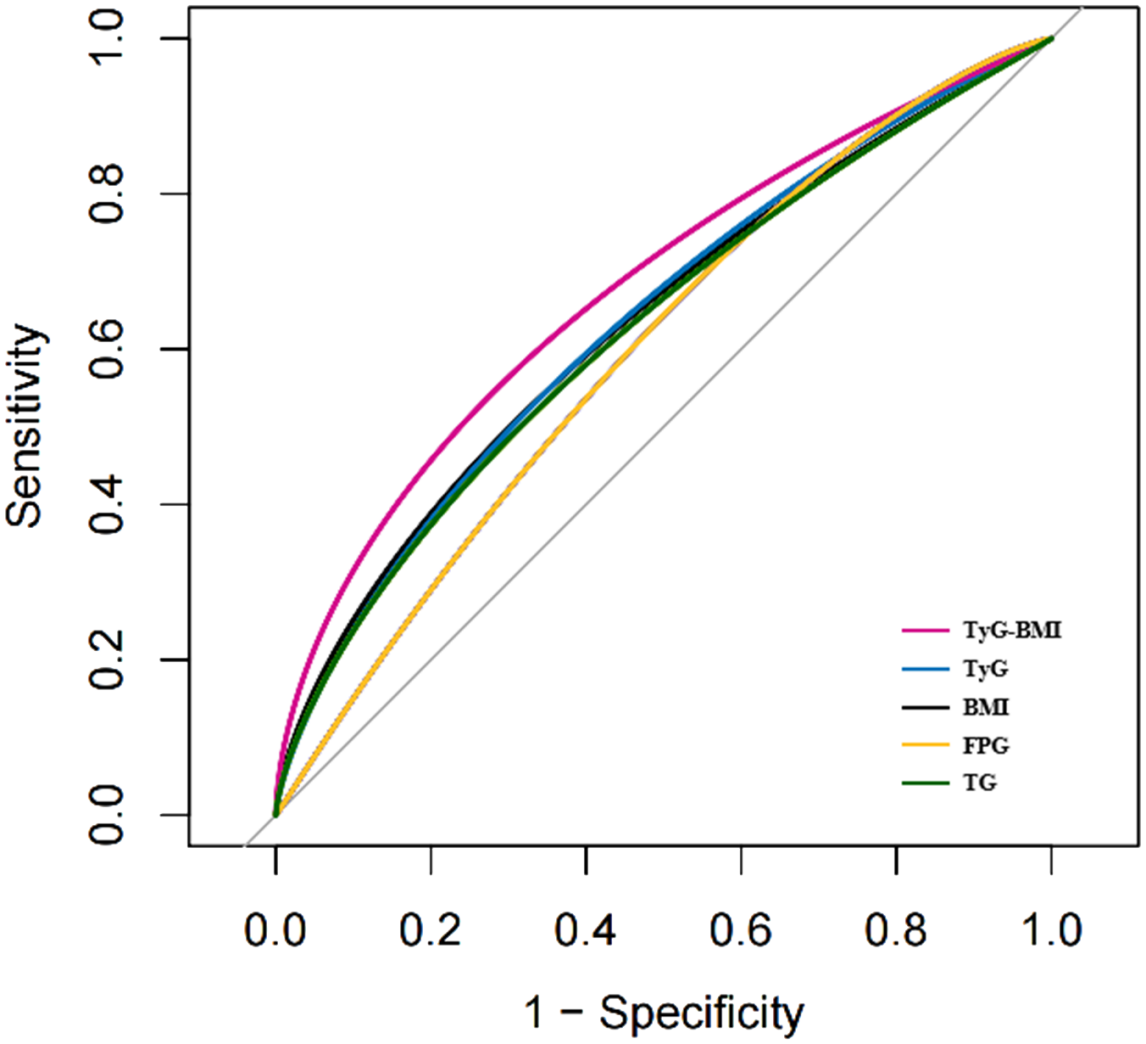
The ROC curves of TyG-BMI and its main components for diagnosing early-onset T2D. TyG-BMI, triglyceride glucose-body mass index; TyG, triglyceride glucose index; BMI, Body mass index; FPG, Fasting plasma glucose; TG, Triglycerides.

An ROC curve was used to evaluate the diagnostic value of TyG-BMI in patients with early-onset T2D (**Figure 5**). The results showed that the AUC of TyG-BMI was 0.6781, which was higher than that of TyG (AUC = 0.6509), BMI (AUC = 0.6368), FPG(AUC = 0.6097), and TG (AUC = 0.6358), all with *P* < 0.001. The optimal cutoff value of TyG-BMI was 254.865, the sensitivity was 74.6%, and the specificity was 53.6% (**Table 5**).

**Table 5:** Diagnostic value of TyG-BMI for early-onset T2D.

Variable	AUC	95%CI low	95%CI upper	Best threshold	Specificity	Sensitivity
TyG-BMI	0.6781	0.645	0.711	254.865	0.746	0.536
TyG	0.6509	0.617	0.685	9.625	0.616	0.622
BMI	0.6368	0.603	0.671	26.295	0.776	0.446
FPG	0.6097	0.575	0.645	9.475	0.496	0.704
TG	0.6358	0.602	0.670	1.975	0.636	0.580

AUC, area under the curve; OR, odds ratios; T2D, type 2 diabetes; TyG-BMI, triglyceride glucose-body mass index; TyG, triglyceride glucose index; BMI, Body mass index; FPG, Fasting plasma glucose; TG, Triglycerides.

## 4 Discussion

In this cross-sectional study based on the Chinese population, we found that patients with early- onset T2D had higher levels of HbA_1c_, FPG, TC, TG, and SUA and more severe overweight/obesity, metabolic disorders, and IR at disease onset. In addition, we found a nonlinear dose-response relationship between TyG-BMI and newly diagnosed early-onset T2D, which was more significant in young men, overweight patients, and patients with a family history of diabetes, fatty liver disease, and hypertension.

Since the early 21st century, clinical studies from different countries have reported on the features of early-onset T2D, and the results have been compared with those of late-onset T2D. A cross-sectional retrospective study in South Korea concluded that patients with early-onset T2D were characterized by higher blood glucose levels, more family history of diabetes, early-onset of microalbuminuria, and insulin therapy as initial treatment (31). The Joint Asia Diabetes Evaluation (JADE) cohort study, conducted at 204 hospitals in Asia, showed that people with early-onset T2D had a more extensive family history of diabetes, as well as higher BMI, HbA_1c_, and LDL-C levels (7). An investigation based on 30-year longitudinal data from the CARDIA study showed that compared with patients with late-onset T2D, patients with early-onset T2D had significantly worse overall and central obesity in early adulthood, with adverse metabolic profiles years before diabetes (32). In this study, we also found similar features, and severe metabolic disorders in patients with early-onset T2D may be an early manifestation of IR (10, 33, 34).

Studies have shown that traditional risk factors for diabetes are amplified in younger patients, and obesity remains the dominant factor (3, 35). In this study, after adjusting for confounders, we found that male sex, family history of diabetes, and high levels of BMI, HbA_1c_, TG, LDL-C, and SUA may have positive effects on the onset of early-onset T2D. Among these factors, hyperglycemia, obesity, and atherogenic dyslipidemia (i.e., elevated TG and decreased HDL-C levels) are the main components of metabolic syndrome. A decade of follow-up in the Tehran Lipid and Glucose Study found that among Tehran adults, both men and women with metabolic syndrome had a 2.9 times higher risk of developing T2D than those without metabolic syndrome; high levels of FPG, TG, and stable low levels of HDL-C increased the risk of T2D (36). We found that people with a family history of diabetes were 1.638 times more likely to develop early-onset T2D than those with late-onset T2D. Genetic predisposition is highly associated with early-onset T2D and has been demonstrated in previous studies. When a first-degree relative has diabetes, the offspring are at high risk of impaired fasting glucose levels, even without obesity. A study in an Iranian population showed that a family history of diabetes and elevated BMI alone increased the lifetime risk of diabetes in both men and women, with obese men with a family history of diabetes having a lifetime risk of about 54% higher at age 20 compared to normal-weight men without a family history of diabetes (37).

In this study, we observed a J-type nonlinear association between TyG-BMI and the risk of early-onset T2D. Although the underlying mechanism of the association between TyG-BMI and early-onset T2D is unclear, based on the clinical characteristics of patients with early-onset T2D, we speculate that severe IR and overweight/obesity may be important causes of early-onset in patients at high risk for T2D. TyG-BMI can simultaneously capture BMI, blood glucose, and lipid profiles and has emerged as an IR replacement indicator in recent years. Several studies have suggested that TyG-BMI is superior to TyG and some indicators related to lipids and obesity in evaluating IR (29, 38). Two Chinese cohort studies respectively found that TyG-BMI was positively associated with prediabetes and diabetes risk, and the risk of developing TyG-BMI related diabetes (prediabetes) was significantly increased in non-obese people and in young and middle-aged people (25, 26). We also found that the J-type relationship between TyG-BMI and early- onset T2D was more significant in young male population, and the risk of early-onset T2D increased more significantly with the slope of the TyG-BMI increase curve, which was different from the higher average TyG-BMI level and higher risk of pre-diabetes in the female population in the study by ZOU et al. (26) Another 5-year cohort study showed a positive, nonlinear relationship between TyG-BMI and diabetes risk in Chinese prediabetic patients, and this positive relationship was stronger in participants < 50 years of age (27). A number of previous studies have reported that TyG and BMI are independently associated with increased risk of diabetes in young people. For example, Ali et al. investigated the incidence of T1D and T2D in UK children and young adults under 25 years of age in relation to high BMI, showing that obesity leads to a four-fold increased risk of developing T2D in individuals (15). MA et al. analyzed data from the Chinese Health Screening project and found that elevated TyG index was independently associated with an increased risk of diabetes in individuals and was more sensitive in individuals younger than 40 years and without hypertension and obesity (39). Whether TyG-BMI, a combined measure of TyG and BMI, improves predictive power is unknown. We explored this issue using ROC curves, and the results showed that TyG-BMI was superior to its main components (TyG, BMI, FPG, and TG) in the diagnosis of early-onset T2D, with moderate AUC value but low specificity. The value of TyG-BMI in the diagnosis of early-onset T2D is limited, and in clinical application, hyperglycemia is still the only criterion for the diagnosis of T2D. Its predictive value needs to be confirmed by large-scale cohort studies. However, the diagnostic or predictive value of TyG-BMI in diabetes may vary among populations. Robinson et al. evaluated the predictive power of 11 obesity- and lipid-related parameters, including TyG, BMI, and TyG-BMI, in a Colombian elderly population at high risk of prediabetes, and showed that the TyG index had the best discernability in predicting prediabetes in older adults (40). In this study, the superior performance of TyG-BMI also supports that obesity may be an important reason for promoting the early onset of T2D.

In the subgroup analysis, we found that coexisting factors such as male sex, family history of diabetes, overweight, fatty liver, and hypertension with TyG-BMI increased the risk of early-onset T2D. Epidemiological studies in several countries have reported that being overweight/obese in children and adolescents is associated with an increased incidence of T2D in early adulthood (7, 16, 41–44). Results from a nationwide population study in Israel showed an interaction between BMI, sex, and adult T2D events. In contrast to this study, several studies have found a stronger association between high BMI in children and adults with T2D in women, all of which were conducted in minors (41, 45, 46). For people with high TyG-BMI, especially young men, combined with the above high-risk factors, strict control of blood pressure, effective weight loss, and treatment of fatty liver to reduce the risk of early-onset T2D.

This cross-sectional study is the first to explore the association between TyG-BMI and early- onset T2D, analyze the risk factors for early-onset T2D from a new perspective, and provide new ideas for the prevention and control of diabetes in high-risk populations. This study had several limitations. First, since the study population was from the same tertiary hospital and the sample size was relatively small, the generalizability was limited; therefore, it is necessary to verify the conclusions in a larger and more diverse population. Second, despite adjusting for common confounding variables, the logistic regression analysis did not completely eliminate the differences between the groups. Third, it would be valuable to further observe the association between TyG-BMI and early-onset T2D during follow-up to evaluate the accuracy of its long-term prediction.

## 5 Conclusion

Patients with early-onset T2D are characterized by severe IR, metabolic disorders, and being overweight/obese and an increase in TyG-BMI is independently associated with an increased risk of early-onset T2D.

## Data availability statement

The raw data supporting the conclusions of this article will be made available by the authors, without undue reservation.

## Ethics statement

The studies involving humans were approved by The Ethics Committee of the Second Affiliated Hospital of Nanchang University. The studies were conducted in accordance with the local legislation and institutional requirements. The participants provided their written informed consent to participate in this study.

## Author contributions

YJ: Data curation, Investigation, Methodology, Software, Writing – original draft. XL: Supervision, Validation, Writing – review & editing.
